# Materials Selection and Design Options Analysis for a Centrifugal Fan Impeller in a Horizontal Conveyor Dryer

**DOI:** 10.3390/ma14216696

**Published:** 2021-11-07

**Authors:** Andrii Zinchenko, Kostiantyn Baiul, Pavlo Krot, Aleksander Khudyakov, Sergii Vashchenko, Aleksandra Banasiewicz, Adam Wróblewski

**Affiliations:** 1 Institute of Transport Systems and Technologies, National Academy of Sciences of Ukraine, 49000 Dnipro, Ukraine; zina@dsu.dp.ua; 2 Z.I. Nekrasov Iron and Steel Institute, National Academy of Sciences of Ukraine, 49000 Dnipro, Ukraine; baiulkonstantin@gmail.com (K.B.); khudyakovsashko@gmail.com (A.K.); sergeyvaschenko.sv@gmail.com (S.V.); 3 Faculty of Geoengineering, Mining and Geology, Wrocław University of Science and Technology, 50-370 Wrocław, Poland; aleksandra.banasiewicz@pwr.edu.pl (A.B.); adam.wroblewski@pwr.edu.pl (A.W.)

**Keywords:** horizontal conveyor dryer, raw materials, centrifugal fan, impeller, FEM

## Abstract

Comparative strength analysis of two popular options of the radial centrifugal fan impeller design used in horizontal conveyor dryer for fine-grained raw materials is presented. Three types of materials for impeller manufacturing—ASTM A36 steel, Hardox 450 steel and aluminium alloy 6061-T6 are considered. The finite element method (FEM) has been used to investigate stresses and deformations of the impeller within the operational speed range. Analysis shows that the better design is the impeller made of Hardox 450 steel with a central disk. Although the maximum stress is slightly higher in the blades slot for central disk fitting for this design option, it has greatly reduced stresses in contact edges with two other disks (by 22–38%) and blades bending deformation (by 51%). For this design, the maximum operational rotation speed is 1135 min${}^{-1}$ according to the yield strength with a 15% safety factor, while for basic design, it is 1225 min${}^{-1}$. The rational choice of material depends on maximum value of the yield stress to density ratio as well as taking into account the operating conditions and required fan performance. Recommendations for manufacturing the centrifugal fan impeller related to chosen material are given.

## 1. Introduction

In the mining, metallurgical, chemical and agricultural industries, precise humidity control of raw material is highly important, in both the intake and processing stages. The inaccurate or moist raw material may be quite expensive when it is purchased by weight. To ensure the required moisture content in raw materials, the various continuous drying units for lumpy, granular and fibrous materials are used [1,2,3,4,5].

**Figure 1:**
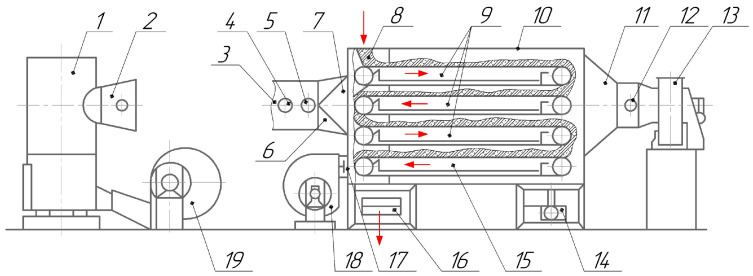
Functional diagram of a horizontal dryer: 1—fire-chamber; 2, 5—thermometers; 3, 11—pipelines; 4, 7, 12, 17—butterfly valves; 6—diffuser; 8—feed hopper; 9, 15—conveyor belts; 10—drying chamber; 13, 18, 19—radial centrifugal fans; 14—reducer; 16—discharge conveyor.

A general view of the design and principle of operation of a horizontal conveyor dryer is presented in Figure 1. This type of equipment is used for low-temperature drying (+40⋯+250 ${}^{{}^{\circ}}$C) of bulk materials requiring careful transportation during moisture removal. Such operation, for example, is included in briquetting various types of fine-grained raw materials (coal sludge of mines, coke breeze, rolling mill scale, ferroalloy screenings, etc.) produced on roller presses [6,7,8,9,10]. In the process of drying, excessive moisture is evaporated and briquettes are hardened.

### Impeller Design Background and Research Tasks

**Figure 2:**
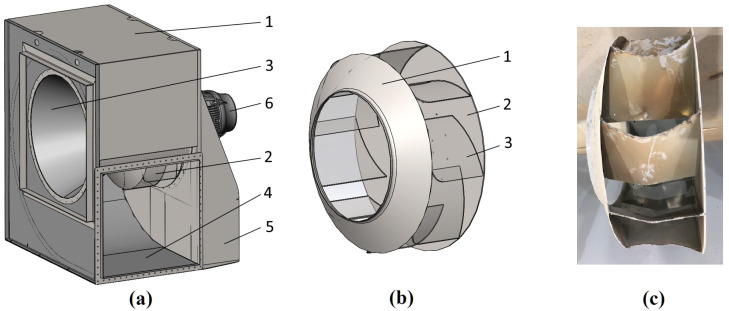
Radial centrifugal fan of a horizontal dryer (**a**): 1—casing; 2—impeller; 3—inlet pipe; 4—outlet pipe; 5—bed; 6—electric motor; impeller basic design (**b**): 1—front disk (shroud); 2—rear disk (hub); 3—blade; impeller blades with wear and deformations (**c**).

The radial centrifugal blower (Figure 2a) is one of the core components of horizontal conveyor dryers. This unit belongs to the separate class of dynamic action machines designed to compress, supply or exhaust air or other gases [11,12,13]. The main rotating element is the impeller (Figure 2b), connected to the driving electric motor by intermediate flange coupling. The impeller is subjected to intensive wear and deformations of its blades (Figure 2c).

According to API STD 673, 2.5.3 [14], the shrouds, back and centre plates of the welded fan impeller shall normally be of one-piece construction but can be joined by full strength penetration butt welds.

A large number of works focused on the study and analysis of design, used materials, parameters and operating modes of centrifugal fans and pumps, including the impellers similar by design shown in Figure 2b are known.

Paper [15] presents the behaviour of an impeller installed on a centrifugal pump. The pump vibration signals under normal conditions with an undamaged impeller and the pump vibration signal with a damaged impeller were compared. Additionally, the signal from a clogged impeller was measured. The results show that the investigated types of features show a corresponding relationship depending on the defects.

In [16], modal analysis and assessment of the pre-stressed state of the impeller of a centrifugal pump for blades of different thicknesses using the finite element method (FEM) were carried out. Results of the numerical simulation were compared with experimental data. The proportional reduction of vibration level with increasing the blade thickness has been shown.

Similar studies are described in [17] where authors carried out a theoretical and experimental analysis of the stress–strain state of the impeller of a centrifugal fan. The theoretical calculation of the stress distribution field was obtained using the FEM, the results of which were confirmed experimentally. The studies were conducted for the basic design of an impeller and for the design with additional rings that add rigidity.

The modal analysis of the impeller carried out in [18,19] has confirmed the negative effect of cyclic loads during the impeller rotation on the strength and durability of the structure under the study.

In [20], a static and modal analysis of an impeller made of three different materials—steel, composite, and aluminium—was carried out. It was found that the natural frequency for the composite material was very low compared to steel and aluminium; however, the deformation was greater compared to steel and aluminium.

A comparative analysis of the stress–strain state and modal analysis of impellers made of composite material and aluminium carried out in [21], showed that the use of composite material results in decreasing the deformations and stresses.

An integrated approach to modelling industrial fans, taking into account specific technological conditions, has been presented in [22], allowing to simulate the performance at peak pressure for conditions of maximum efficiency of operating parameters and to make a prediction of the nature of impeller erosion as well.

The analysis conducted in [23] of the severe failure of the fan impeller rotor made of S690Q unalloyed heat-treated high strength steel showed the influence of the welding technological regimes on the high-strength steels joining that caused imbalance and overall structural instability under high centrifugal force.

Failures of the shrouded fan blade caused by uneven contact inducing excessive stress at the contact edge are considered in [24]. An effective method to reduce contact stress is to increase the tightness of the contact surface and include a pre-deformation.

The catastrophic failure of the main blower of a petrochemical plant boiler is investigated in [25] where the rotor was subjected to strong unbalanced forces because of one or more blades breakage due to excessive rotating speed.

Using FEM, the maximum stress at the root of the blade is calculated in [26]. According to the developed model of fatigue life prediction, reducing the stress concentration is an effective way to increase the fatigue life of fan blades.

In the work [27], the authors describe the fan impeller, in which static strength and modal analysis using the finite element method are applied for optimal design instead of the traditional calculation method of strength and stiffness.

Based on Computational Fluid Dynamics (CFD) a comprehensive model accounting for all aerodynamic losses for numerical optimization to redesign the fan blades, inlet duct, and shroud of the impeller is developed in [28].

CFD simulations are also utilized to improve energy efficiency in mine ventilation fans. In [29], the examination of fan performance has been done through the analysis on flap-adjustment and leading-adjustment in aerodynamic performances. This included the angle of blades, total pressure, efficiency, system efficiency, adjustment efficiency, and energy-saving rate.

In [30], a study on the selection of an aerofoil blade profile has been conducted. Simulations were performed on six typical aerofoil sections at different angles of attack, ranging from 0 to 21. and at Reynolds number Re = 3 × 10${}^{6}$, and various aerodynamic parameters.

The CFD investigation on blades profile has been presented as well in [31]. Two types of profiles, namely Forward and Backward Skewed blades, were analysed in order to compare the static pressure, flow rate, flow coefficient, and pressure coefficient generated by the fan and thus determine their efficiencies.

A study on blades optimization design is presented in [32]. The emphasis has been put on topology optimization with FEM. The results imply the possibility to reduce the mass of blades by 60% in comparison to monolithic blades.

Fatigue, as shown in the paper [33], is the main type of damage to impellers and blades. Fatigue damage contributes to significant economic losses for companies and society, but also endangers human lives.

The modal and fatigue analysis of a centrifugal fan impeller is carried out in [34] to obtain the natural frequencies and mode shapes. Additionally, the fatigue analysis is performed to estimate the fatigue life, safety factor and damage risk.

Experimental and FEM stress analysis made in [17] has shown the high complexity of stress patterns in impeller parts, which can be reduced using additional rings on the blades. The authors concluded that stiffening rings located at the nose and tail of the blade, both reduce the stress.

The results of [35] showed that the blade trailing edge and the shroud plate have maximum von Mises stresses, which exceed the yield limit of the steel. The thickness effect of each part on the stress distribution in the impeller was determined. The shroud plate is a critical part and has a larger effect on the stress distribution than other parts.

Authors of the paper [36] concluded that centrifugal fans design is still based on various empirical and semi-empirical rules used by manufacturers.

Summarizing the above-mentioned studies, the main directions in research of the centrifugal fan impellers are the analysis of the influence of geometric parameters, properties of the used material and operating modes on the strength and durability as well as on the level of vibration and noise in operation [37].

The motivation for the current research was the quick deterioration of the impeller and the need to increase the productivity of the centrifugal fan (speed of rotation) with simultaneous minimisation of impeller manufacturing and further maintenance costs. The novelty of the represented approach is a combination of FEM modelling of dynamical loads in the elements of impeller and application of design criterion, which includes mechanical properties of materials.

In the present paper, authors study the influence of design schemes, design parameters and material properties of a dryer centrifugal fan impeller on its ultimate strength at various speeds of driving motor rotation. At the same time, improving the performance and ensuring the strength of the impeller within rotation speed-up are the practical tasks of possible impeller modernization regarding specific drying units. The relevance of this study is confirmed by increased requirements for the reliability of units and assemblies of metallurgical and mining equipment operating under complex loading conditions [38,39] and increased speeds of rotation at high risk of failures.

## 2. Impeller FEM Simulation and Design Options Analysis

The design of centrifugal fans is an interdisciplinary process, including aerodynamics, thermodynamics in case of hot air flow, stress analysis, modal analysis of resonances, the selection of materials, and the requirements for the manufacturing process.

The strength of the impeller is one of the key factors of reliability of the dryer centrifugal fan, due to the large overall dimensions of this element, big weight, increased rotation speed (up to 1500 min${}^{-1}$) and significant centrifugal loading during operation. Additional vibration loads during fan operation are caused not only by assembly inaccuracies, but also by aerodynamic processes in the flow path. Furthermore, high vibrations create additional loads on structural parts and increase the noise level.

Such fans usually undergo initial factory balancing to eliminate the imbalance as the key factor of increased vibration. Unfortunately, during operation, the blades become dirty and additional dynamic loads difficult to evaluate experimentally occur. Therefore, to analyse stresses under loading as accurately as possible, the finite element modelling of the aggregate is applied.

There are two main methods for improving the specific strength parameters of the impeller: (1) changing the geometric parameters of the structure; (2) using specific materials and methods of assembling parts. In this work, both ways are considered.

### 2.1. Choosing Materials and Method for Connecting Blades in Impeller Assembly

Due to a special dryer configuration where the blower is installed before the burning chamber as shown in Figure 1, the impeller parts are not experiencing high temperatures. Therefore, a wider range of materials may be used for manufacturing the impeller including aluminium alloys and composite materials.

The following materials, popular among manufacturers, such as ASTM A36 steel, Hardox 450 special steel and 6061-T6 aluminium alloy were adopted for carrying out computational and analytical studies. The main physical and mechanical characteristics of these materials are shown in Table 1.

ASTM A36 is a commonly known and cheap structural low-alloy steel, easy to cut, easy to weld and deform, and can be the first choice material in terms of reducing the cost of the fan impeller. It was found in [43] that for stud welding of 3 mm thick ASTM A36 steel, the optimal parameters are as follows: main-current time 25–35 ms and pre-current time 20–60 ms.

Hardox 450 steel is a high strength material with high impact toughness that retains its physical and mechanical properties over a wide temperature range. This steel has high wear resistance, good bending and stamping, and good welding ability. The results obtained in [44] showed a large influence of the parameters of the preliminary heat treatment on the selected mechanical properties of the welded joints of this steel. Although the relatively high price, the combination of these properties makes it the good choice for centrifugal fan impeller manufacture with considerably increased strength and service life.

Aluminium alloy 6061-T6 is a dispersion-hardened alloy with good ability to deformation, welding, machining and thermal hardening. This aluminium alloy is quite suitable for the manufacture of fan impellers operating in the absence of high temperatures, i.e., in workshops at ambient temperatures, or open spaces at moderate temperatures. An impeller made of aluminium alloy will have a significantly lighter weight with a positive effect on its performance. Authors in [45] investigated the microstructure and properties of welded material (WM) and base material (BM) of aluminium alloy 6061-T6. They showed that the strength of WM was less sensitive to strain rate compared with BM.

It is important to note that further analysis is based on the assumption that all parts of the impeller are joined properly by appropriate methods of welding.

### 2.2. Design Options and Parameters of Impellers

**Figure 3:**
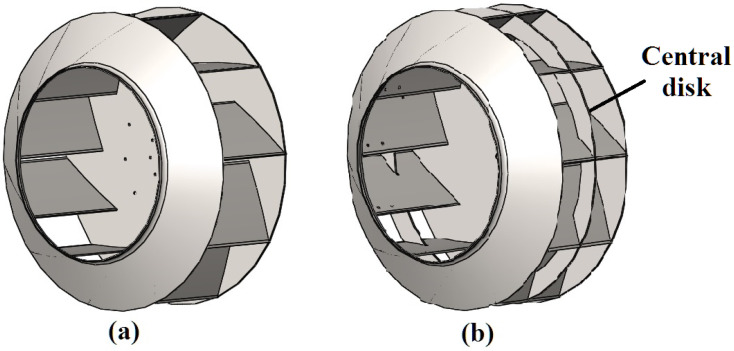
Analysed design options of impeller: (**a**) the impeller of the basic design; and (**b**) impeller with an additional central disk.

In this study, two design options of the impeller are considered (see Figure 3). The difference between them is the presence of an additional central disk between the hub and shroud in the second version (Figure 3b). This modification is quite popular among manufacturers of impellers and is aimed at increasing the rigidity of the structure and reducing deformations that occur in the impeller during operation.

The overall dimensions of both impeller options are identical and are as follows: outer diameter—1105 mm, width—480 mm. The sheet metal thickness of the impeller parts is 6.35 mm (1/4${}^{\prime \prime }$). The masses of impellers made from different materials are given in Table 2. Adding a central disk significantly (by 25%) increases the mass of the impeller, but makes it more rigid with less bending deformation of the blades.

The number of rotor blades for both design options is 10. The number and position of blades in the structure of the impeller, as well as geometric parameters of curvilinear blade profile, were pre-defined to obtain the required fan performances.

## 3. Influence of Rotation Frequency on the Stress-Strain State of the Impeller

The operating rotation frequency of the drying units’ impellers is, as a rule, within the range of 1200–1500 min${}^{-1}$ and is changing by the motor speed regulator. Therefore, for this study, the range of impeller rotation frequencies from 500 to 1500 min${}^{-1}$ was adopted.

Stresses in a rotating impeller are created by centrifugal forces, as well as a pressure drop across the surface of the blades. In this case, the airflow force effect on the impeller is negligible in comparison with the centrifugal forces from its rotation [46,47,48]. To verify numerical values concerning the problem solved in this work, the aerodynamic studies of airflow (Computational Fluid Dynamics) were carried out. It was found that the force effect of the airflow on the impeller does not exceed 2% of the total force effect, and the resulting pressure forces unload the blades. Consequently, to simplify the subsequent computational and analytical studies, it is quite acceptable to disregard the effect of the air pressure. Further analysis of the stress–strain state of the studied impeller models caused by centrifugal forces without taking into account the airflow force effect was carried out using the FEM software Ansys 2021 R2.

When creating a three-dimensional model for calculations, the properties of materials were set and welded joints assumed as zero-radius at the joints of impeller parts were taken into account. At the same time, it is assumed that the properties of the weld material are similar to those of the base metal of the parts. This assumption is justified by the fact that for the materials adopted in the work (see in Table 1), subject to the recommendations for performing welding, the strength parameters of the weld, as a rule, are higher than of the base metal.

**Figure 4:**
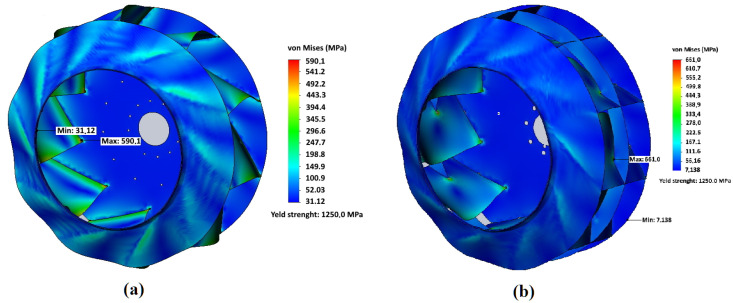
Von Mises stress distribution in the impeller: (**a**) impeller of the basic design, and (**b**) impeller with central disk; impeller material—Hardox 450 steel; rotation frequency—1000 min${}^{-1}$.

**Figure 5:**
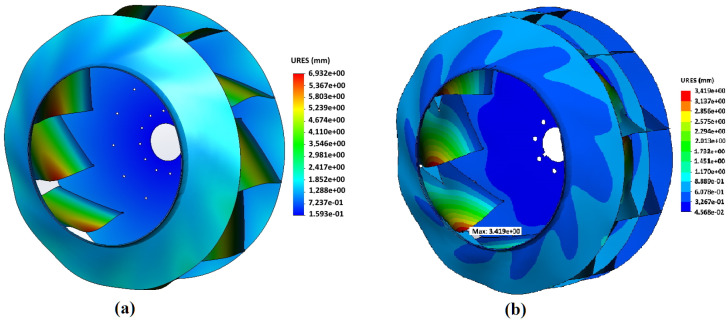
Distribution of deformations in the impeller: (**a**) impeller of the basic design, and (**b**) impeller with central disk; impeller material—Hardox 450 steel; rotation frequency—1000 min${}^{-1}$.

In Figure 4 and Figure 5, an example of the results of calculating the stress–strain state of impellers made of Hardox450 steel during operation with rotation speed 1000 min${}^{-1}$ is shown for both designs of the impeller adopted in the work—the basic one and with an additional central disk. For both variants of impeller designs, significant stresses arise at the points where blades are attached to the front and rear disks in the areas close to the impeller axis of rotation. In the basic design of the impeller, the maximum stresses of 590.1 MPa are generated in these sections. For an impeller with an additional central disk, the maximum stress values arise in the area where the additional central disk rests on the blade bodies—661.0 MPa. Concerning deformations, for both design options, the maximum values drop on the sections of the middle of the blades from the side of the impeller rotation axis. For an impeller of the basic design, the maximum deformations are 6.93 mm, and for an impeller with an additional central disk, they are 3.42 mm that is 51% less and confirms the initial assumption of a higher rigidity of the modified design.

**Figure 6:**
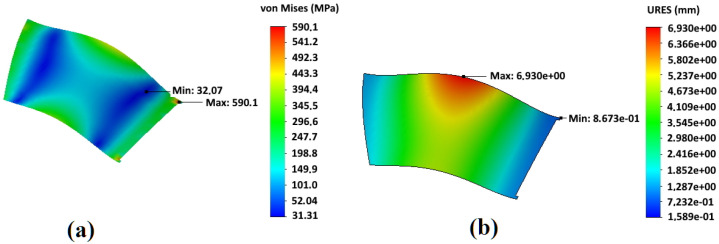
Stress–strain state of an impeller blade of a basic design: (**a**) stresses; (**b**) deformations.

**Figure 7:**
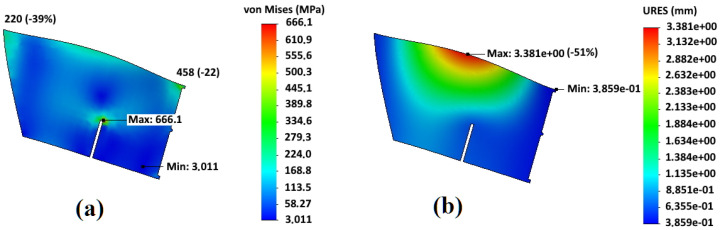
Stress–strain state of an impeller blade with an additional central disk: (**a**) stresses; (**b**) deformations.

In Figure 6 and Figure 7, the results of calculating the stress–strain state of the blades of an impeller of the basic design and an impeller with an additional central disk are shown. They made it clear that the areas of maximum deformations do not correspond to the areas of maximum stresses that are at the junction of the blades with the rear disk. In this case, the sections with the maximum values of stresses correspond to the minimum value of deformations.

Analysis of the stress–strain state of two considered impeller designs shows that the basic design is preferable for the minimization of stresses, and design with an additional central disk is preferable to reduce bending deformations of the blades.

In Figure 7, points of higher stress appear near the top of the slot for central disk adjustment. While the maximum stress at the junction point with the rear disk is 458 MPa, that is 22% less than for basic design (590 MPa). The corresponding value at the blade edge in front disk contact is even more reduced from 360 MPa to 220 MPa (−38%). The bending deformations at the middle of blades are greatly reduced by more than two times. Hence, the central disk plays an important role in fatigue resistance, especially when the dust is sticking to the blades and increases inertial bending forces. However, the slightly higher (by 10–12%) stresses are observed in the blades slot for central disk fitting, which can be managed by the appropriate technical arrangements and welding regimes as described below.

**Figure 8:**
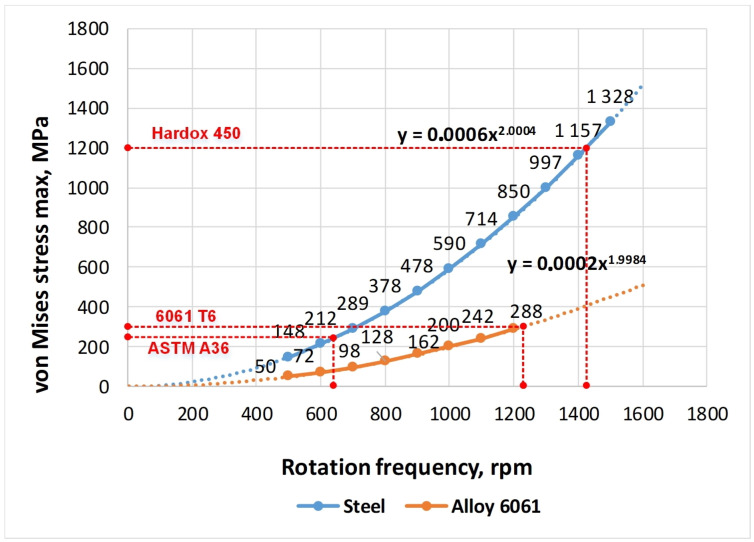
Dependence of the maximum stress in an impeller of the basic design on the rotational speed for steel (ASTM A36, Hardox 450) and aluminium alloy 6061-T6.

**Figure 9:**
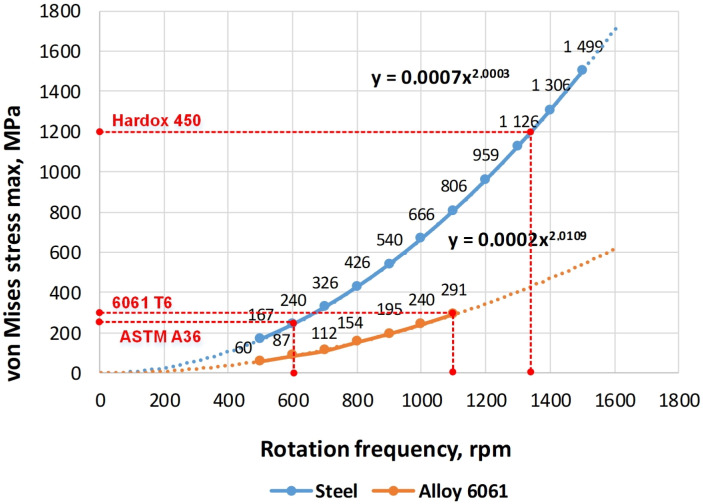
Dependence of the maximum stress in an impeller with a central disk on the rotational speed for steel (ASTM A36, Hardox 450) and aluminium alloy 6061-T6.

To find the limiting values of the rotational speed of the investigated impellers, the calculations of the maximum values of the stresses of the elements in the range of rotation frequencies from 500 min${}^{-1}$ to 1500 min${}^{-1}$ impeller were carried out. These dependencies are shown in Figure 8 and Figure 9. The dashed lines mark the boundaries of the maximum permissible rotational speeds of the impellers according to the condition of reaching the yield point at the maximum loadings of the structure.

The graphs of stresses in the impeller on the rotational speed for ASTM A36 and Hardox 450 steel coincide because their values of density, elastic modulus and Poisson’s ratio are the same. The difference between these materials lies in the magnitude of the yield strength and strength itself. Logically, the graphs for the aluminium alloy differ significantly from the graphs for steel. This is because of the essential difference in the physical and mechanical characteristics of these materials.

As noted before, the strength of the impeller welds, as a rule, turns out to be greater than the strength of the base metal of the structure. Therefore, when analysing the strength of the impellers, the ultimate strength and yield strength of the base material were taken into account. However, it is generally known that the weakest part of the welded structure is the transition from the welded seam to the base metal, which can be quite brittle if the welding regimes are violated. Taking this fact into account, it was assumed that, for the safe operation of the impeller, the maximum permissible frequency of its rotation during operation should be 15% less than the maximum possible value according to the strength conditions of this structure.

Using the data shown in Figure 8 and Figure 9 and the adopted reduction of the permissible rotation frequency by 15%, the boundary indicators of the operating modes of the fan impellers are determined; their values are presented in Table 3 below. To summarise the results of the calculations, the yield stress to density ratio is introduced as the criterion for three materials comparison. The maximum value corresponds to Hardox 450 and the lowest value to ASTM A36 steel although it has greater yield stress than 6061-T6 alloy.

The graphs in Figure 8 and Figure 9 and data in Table 3, show that at equal values of the rotation speed, impellers made of Hardox 450 steel achieve the best strength results. Thus, their maximum permissible rotational speed is 46–47% higher than for impellers made of ASTM A36 steel and by 16–18% is higher than the value of aluminium alloy 6061-T6.

## 4. Recommendations for the Manufacture of Impellers

According to the abovementioned research results, wear-resistant steel Hardox 450 is recommended for the manufacture of impeller parts. This material is known to be low–alloyed and low-carbon, and therefore has very good welding characteristics. The main properties of Hardox steels are given in Table 4. The Hardox 450 among the other grades has the maximum value of impact toughness, although some grades have large tensile strengths (Hardox 500, 550, 600). Impact strength is important for the impeller design because it is an enough large structure, which rotates at a high frequency (up to 1500 rpm) and fracture damage is very dangerous for the whole centrifugal fan. The advantage of using steel with a higher impact toughness in a fan impeller allows having a greater resistance to shock loads during abrupt starts or stops, hitting foreign objects, etc.

(1) Brinell hardness, HBW, according to EN ISO 6506-1, on a milled surface 0.5–3.0 mm below surface. At least one test specimen per heat and 40 tons.
(2) Min. Impact energy (J) for transverse tests Charpy V 10 × 10 mm test specimen. Impact testing according to ISO EN 148 per heat and thickness group. Average of three tests. Single value minimum 70% of specified average.
(3) Longitudinal test, typical impact energy, Charpy V 10 × 10 mm test specimen. Impact toughness measured upon agreement. For thicknesses between 3 mm and 11.9 mm, sub-size Charpy V-specimens are used. Impact testing according to ISO EN 148 per heat and thickness group. Average of three tests.

However, the difference in thermophysical properties of Hardox steels [51] depending on parts geometry requires some special modes of welding to avoid cracks and degradation of mechanical properties. The low thermal conductivity and a high coefficient of linear expansion, characteristic of alloy steels, cause, under all other equal conditions (welding method, sheet thickness, etc.), the expansion of the near the weld zones heated to different temperatures. An increase in the deformation of the metal leads to distortion of structures. The greater the coefficient of linear expansion and the higher the heating temperature, the greater deformation the metal will experience during heating and cooling. Therefore, welding methods and modes should be used, with a calculated thermal energy input per seam length to provide the required cooling time. Based on SSAB manuals of Hardox steels welding [52], some practical rules can be given for impeller manufacturing.

Usually, it is recommended to preheat the parts to be welded to certain temperatures to prevent the formation of cold cracks (so-called hydrogen cracking). Considering the less than 20 mm thickness of the impeller parts, it is possible to weld sections without preheating in production facilities. The impeller parts made of Hardox 450 steel can be welded without preheating using welding electrodes E7018 or wires 309L, ER70S-6.

To increase the fatigue strength of the impeller welds, it is recommended to use heat treatment methods to reduce the concentration of residual stresses in the welded seams. For example, preheating of a newly made weld seam facilitates the release of hydrogen from steel and improves the quality of the metal. The post-weld temperature should be the same as the preheat temperature. The specific holding time should be at least 5 min for every 5 mm of sheet thickness, while the total holding time should be at least 1 h. It is reported in [53] that post-welding heat treatment of parts made of Hardox 600 steel can greatly improve the mechanical properties. Although, not every workshop has the facilities for heat treatment of large-scale structures.

High-strength steel sheets are often supplied with a primer coat and this can adversely affect the quality of the weld. However, the important condition is that the surfaces before welding should be completely clean and dry. Therefore, many manufacturers recommend removing primer from parts to be welded. Hardox 450 has the advantage of being welded directly over the primer. This is facilitated by the low zinc content of the proprietary primer. Although, there is still a slight possibility of increasing the porosity of the weld. The lowest porosity is achieved in the case of semi-automatic welding using filled flux-cored wire. Manual arc welding can also reduce porosity. To obtain a better and more durable joint, it is recommended to remove the primer layer.

Because all welds in the impeller structure are long enough, it is recommended to use a step or reverse step method of positioning the seams. In this variant, the seam is divided along the length into sections of 50–150 mm and welded, if necessary, with the overlap of each next section with the previous one by 10–20 mm. This welding method results in a significant reduction of deformation.

When the thickness of the large-scale impeller’s parts to be welded is more than 4 mm, it is advisable to use multi-layer welding. The seam is filled with several layers. Welding is carried out in short sections, while the joints of the beads should not coincide on different layers. Before applying each layer, the previous one needs to be cleaned to a metallic sheen, this is necessary to increase the density and strength of the welded joint. The legs of the welded seams are recommended 5–10 mm.

It is worth noting that using a slipway can be advantageous to build this structure. In this method, the sheets of the welded metal are clamped over the area with a massive object for heat dissipation, i.e., to minimize the overheated area and keep the sheet in a flat state forcibly until the end of welding. The welded impeller should be placed on a slipway or similar device for the time required to cool down the welds.

In addition to the listed recommendations, the Deep Cryogenic Treatment method [54] can be advised as an efficient heat treatment method to improve microstructure, reduce internal stresses and increase strength without decreasing the impact toughness of parts made of alloy steels.

## 5. Discussion

For impeller design with an additional central disk, the maximum stress at the junction of the blades with the rear disk is 458 MPa that is 22% less than for basic design (590 MPa). The corresponding value at the blade edge in front disk contact is even more reduced from 360 MPa to 220 MPa (−38%). However, calculations of maximum stress at the blade slot for central disk fitting showed that the fan has a maximum rotation speed of 1135 min${}^{-1}$ while the basic design has 1225 min${}^{-1}$ according to the yield strength with a 15% safety factor. The rational choice could be made depending on operating conditions and required performance.

It has been shown that the most effective material option from the considered ones for impeller manufacturing is Hardox 450 steel. The worst strength performance has impeller made from ASTM A36 steel. Intermediate values are corresponding to the impeller made from6061 to T6 aluminium alloy. The advantage of using aluminium alloy is the possibility of a significant lightening of the design while maintaining high strength parameters. Unfortunately, the use of this alloy is limited in practice, because it is possible to make blades from this material only when the fan is installed in a section of the air supply in front of the heating element.

Regarding the sheet metal thickness of impeller elements, as a first approximation, the following considerations can be taken into account. The inertial (centrifugal) force from rotation at each point is proportional to the mass of an individual element and sheet thickness. While the tensile stress at each point is proportional to the force (mass) and inversely proportional to the element section, which linearly depends on the thickness of the sheet. Hence, with increasing thickness, both the numerator and denominator in the stress relation grow linearly and the tensile stress does not change. The bending stress of a thin rectangular clamped plate is inverse proportional to the third degree of thickness. Therefore, until the bending stress is less or comparable to the tension stress, the increasing thickness makes the impeller stiffer and heavier with a higher cost. Since the central disk greatly reduces bending stress, the values of which are less than tensile stresses in our case, the adopted sheet thickness of 6.35 mm is quite suitable for this geometry, weight and rotating speed range of the centrifugal fan.

It is worth noting that Hardox steels have many competitors. For example, Strenx (Weldox) 700; Swebor 400; DILLIDUR 400V; Miilux 400; HARTPLAST 400; Quard 400 etc. These competitors of Hardox steel are similar in most quality indicators and the choice of material is significantly influenced by its availability and price in the regional market. Parameters of each steel (tolerance, flatness) can be found in the specifications of producers.

When designing for enterprises located in North America or Scandinavian countries with a cold climate, the use of Hardox 450 is quite justified. While for the Eastern Europe customers, primarily for economic reasons, the use of locally produced steels is logical and beneficial. The steel grades produced in Ukraine and Poland can also be considered as analogues of SSAB products, e.g., 18HGNMFR. Those steels are inferior to Hardox steels in some parameters, but the difference in price by 1.5…2.0 times and availability in the market make local analogues quite competitive.

## 6. Conclusions

The task of material selection is not so trivial. Besides the yield stress, another crucial factor is the density (specific weight) of material, which determines the total weight of impeller and inertial forces acting on its elements and joints. The best material choice corresponds to the highest yield strength with minimal specific weight. Such ratios are as follows [MPa/(kg/m${}^{3}$)]: ASTM A36—0.032; Hardox 450—0.155; 6061-T6—0.102. Results of calculations illustrate the fact that 6061-T6 alloy provides better performance, i.e., maximum rotation speed in comparison with A36 steel, which has bigger yield stress.

The results of the FEM analysis carried out in this work showed that the basic design of the impeller is favourable from the viewpoint of minimizing local stresses. However, the modified design with the central disk provides greater rigidity and, as a result, the reduction of maximum blades deformations (−51%) in the operating range of rotational speeds with the positive effect on the dynamic parameters and fatigue resistance of the centrifugal fan.

The performed research helps us to better understand the causes of potential failures in centrifugal fan and possible week points in different impeller design options; hence, to improve the reliability of such important rotating equipment engaged in different industrial plants.

Further research is associated with the utilisation of a designed FEM model for simulation of different kinds of failures of impellers disks and blades, to assess their influence on vibration signals registered on the shaft bearing supports by equipment monitoring systems, and to develop methods of its efficient diagnostics as shown in [55,56,57] for other minerals processing machines.

## Figures and Tables

**Table 1:** Properties of materials for impeller manufacturing.

Material	Brinell Hardness, HB	Impact Toughness, KJ/m${}^{2}$	Yield Stress MPa	Ultimate Stress, MPa	Endurance Stress, MPa	Thermal Conductivity, W/(m K)	Coeff. Thermal Expansion, 10${}^{-5}$ 1/K
ASTM A36	160	27 (+20 ${}^{{}^{\circ}}$C)	250	550	160 [40]	50	1.35
Hardox 450	450	50 (−40 ${}^{{}^{\circ}}$C)	1200	1400	460 [41]	21	2.20
6061-T6	95	37 (+30 ${}^{{}^{\circ}}$C)	275	310	230 [42]	167	2.32

**Table 2:** Mass of impellers made of different materials.

Type of Design	ASTM A36, Hardox 450 Steels	Aluminium Alloy 6061-T6
The impeller of basic design	111.13	38.10
Impeller with a central disk	138.41	47.97

**Table 3:** Limiting speeds of impeller with 15% safety factor.

Material	Limiting Speed of Basic Design, min${}^{-1}$	Limiting Speed with Central Disk, min${}^{-1}$	Yield Stress to Density Ratio, MPa/(kg/m${}^{3}$)
ASTM A36	653	605	0.032
Hardox450	1225	1135	0.155
6061-T6	1002	959	0.102

**Table 4:** Properties of Hardox^®^ Wear Plates [49,50].

Hardox^®^	Hardness [HBW] (1)	Yield Strength [MPa]	Impact Energy (−40 ${}^{{}^{\circ}}$C) [J]	Tensile Strength [MPa]	Density [g/cm${}^{3}$]	Weldability
HiTuf	310–370	850	40 (2)	no data	7.72	moderate
400	370–430	1100	45 (3)	1250		
450	390–475	1250	50 (3)	1400		
500	450–540	1400	37 (3)	1550		
550	525–575	1400	30 (3)	1700		
600	550–640	1650	20 (3)	2000		

## Data Availability

Publicly available data on material properties were analyzed in this study.
